# Minimal lower eyelid epicanthoplasty combined with thermal contraction to treat epiblepharon in chinese children

**DOI:** 10.1186/s12886-022-02763-7

**Published:** 2023-01-10

**Authors:** Shoulong Hu, Jingyi Li, Yuebing Lu, Shengnan Zhao, Yi Shao

**Affiliations:** 1 National Center for Children’s Health, MOE Key Laboratory of Major Diseases in Children, Department of Ophthalmology, Beijing Children’s Hospital, Capital Medical University, Beijing, 100045 China; 2 Department of Ophthalmology, Children’s Hospital of Zheng Zhou, Zheng Zhou, 450053 China; 3 Department of Endocrinology, Beijing United Family Hospital, Beijing, China; 4 Department of Ophthalmology, The First Affiliated Hospital of Nanchang University, Jiangxi Province Clinical Ophthalmology Institute, Nanchang, Jiangxi Province 330006 China

**Keywords:** Epiblepharon, Minimal epicanthoplasty, Thermal contraction, Chinese children

## Abstract

**Background:**

To evaluate the clinical efficacy of combined minimal lower eyelid epicanthoplasty and thermal contraction for epiblepharon repair in Chinese children.

**Methods:**

Between January 2017 and August 2020, a single surgeon corrected epiblepharon in Chinese children using minimal lower eyelid epicanthoplasty combined with thermal contraction. First, a minimal epicanthoplasty flap to balance the lower eyelid was made cross the lower eyelid epicanthus, which connected with a 20-mm-long incision 1.5 mm below the lower eyelid margin. After removing the hypertrophic orbicularis oculi muscle running between the lower epicanthal fold and the medial canthal tendon, thermal cauterization was applied to increase lower eyelid rotation by creating adhesions between the lower eyelid retractor and tarsus. The residual medial edge was sutured to the corner of the epicanthus to decrease the lower eyelid epicanthus. The postoperative follow-up ranged from 3 to 24 months. We retrospectively analyzed cases to determine whether this approach decreased the lower eyelid epicanthal fold to equalize the tension of the lower eyelid. The surgical outcomes including the direction of lower eyelid eyelashes, complications, and refractive errors were evaluated.

**Results:**

Data from each eye of 53 Chinese children (29 female, 24 males; mean age: 5.3 ± 2.3 years) who had undergone minimal lower eyelid epicanthoplasty combined with thermal contraction were included. During follow-up, recurrence was observed in just one of the 106 eyes (0.94%), and two eyes (1.89%) showed under-correction. No visible scars formed in the postoperative period. All patients’ parents were satisfied with the cosmetic results and had no serious complaints. The mean astigmatism was significantly reduced by the surgery from 1.82 ± 0.45 diopters (D) preoperatively to 1.43 ± 0.36 D postoperatively (*P* < 0.05).

**Conclusion:**

This surgery is easy to design, time-efficient, and is effective in the correction of epiblepharon. The procedure allows surgeons to achieve good appearance and natural eyelid contour without apparent complications.

## Background

**Fig. 1:**
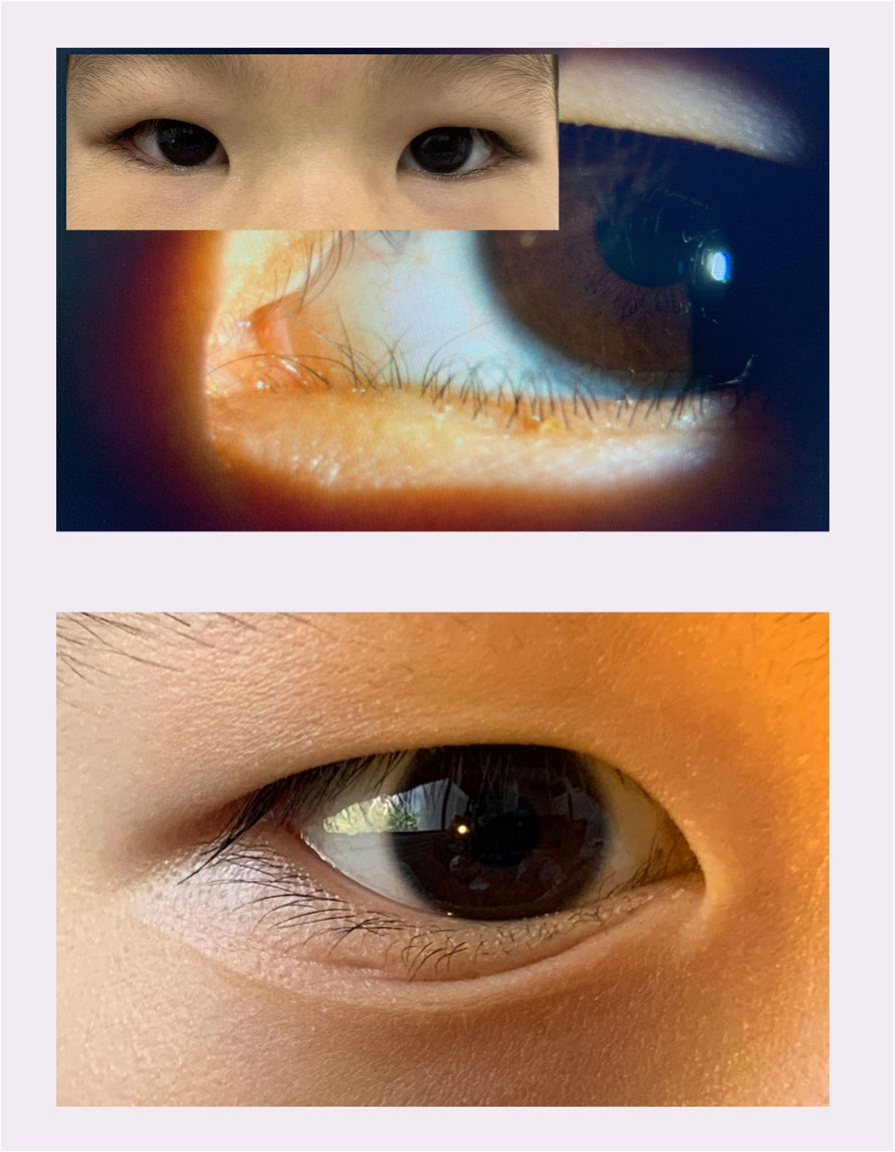
(top) Image of a 5-year-old girl with epicanthus who underwent only Hotz surgery at 3 years. The medial part of the lower eye lid is under-corrected, and the cilia touch the anterior eye surface. (bottom) Image of a 6-year-old boy who was treated with bracing suture methods over 1.5 years. The medial part of the lower eyelid is under-corrected, and a crease is clearly apparent

Epiblepharon frequently occurs in Asian children. This condition is characterized by a medial skin fold extending over the upper and lower eyelid margins, thereby causing the eyelashes to contact the cornea without abnormalities of the tarsal plate and eyelid position [1, 2]. Epiblepharon may spontaneously resolve with age due to facial bone growth and altered balance of eyelid tension. However, surgical repair is indicated in subjects with persistent keratopathy or symptoms of irritation. Several methods have been used for epiblepharon repair, with bracing sutures and the Hotz procedure being the two most popular. Recurrence rates in these procedures can be as high as 29% for bracing sutures and as low as 9% using the modified Hotz procedure [3–5], so it is important for surgeons to understand the factors contributing to high recurrence. The most likely factor may be the presence of epicanthal folds (EFs) in the Asian population [6, 7]; in our clinical experience, bracing sutures or the modified Hotz procedures leave uncorrected epicanthal folds which push the medial eyelashes against the cornea (Fig. 1). In recent years, surgeons who have adopted epicanthoplasty to treat epiblepharon have reached the consensus that the epicanthus not only influences appearance, it also prevents the restoration of lower eyelid balance for patients with epiblepharon. EFs should be considered as a tension imbalance of the lower eyelid which needs to be consistently corrected. Epicanthoplasty for epiblepharon repair may address the tangential cause of the medial EFs. However, few studies on this approach have been conducted in China, and a challenge remains to design surgical techniques that avoid scar formation and changes in appearance. We developed a simple method of minimal lower eyelid epicanthoplasty combined with thermal contraction to eliminate the lower eyelid EF without significantly changing the whole eyelid. Here we describe both the surgical procedure and outcomes in a series of Chinese children.

## Methods

This study is a retrospective analysis of minimal lower epicanthoplasty combined with thermal contraction to correct epiblepharon in children. The procedures were conducted between January 2017 and August 2020 by a single surgeon (author SH) in the Ophthalmology Department of Beijing Children’s Hospital, Capital Medical University, Beijing, China. The indication for surgery was the presence of marked superficial punctate keratitis on fluorescein staining.

The present report follows the Preferred Reporting of Case Series in Surgery (PROCESS) guidelines [8]. We confirm that the current research was carried out in accordance with the principles of the Declaration of Helsinki and applicable local regulations. The study was approved by the Medical Ethics Committee of the First Affiliated Hospital of Nanchang University. Informed consents to participate in the surgery were obtained from the children’s parents.

**Fig. 2:**
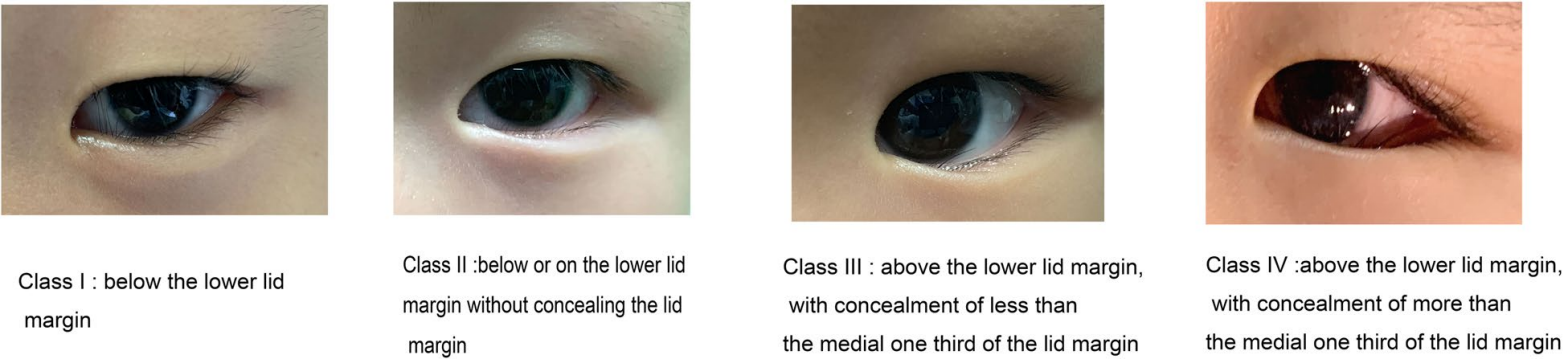
Preoperative classification of lower lid epiblepharon according to the location of the highest line of the lower eyelid skin fold

Lower eyelid epiblepharon severity was graded according to Khwarg’s 1 to 4 classification system based on preoperative skin fold height (Fig. 2) [9].

Surgery was performed under general anesthesia. The following two techniques were used: minimal lower eyelid epicanthoplasty and thermal adjustable rotation of the lid margin. Incision design and the release of tension on the lower epicanthus are the most critical aspects of this surgery.

**Fig. 3:**
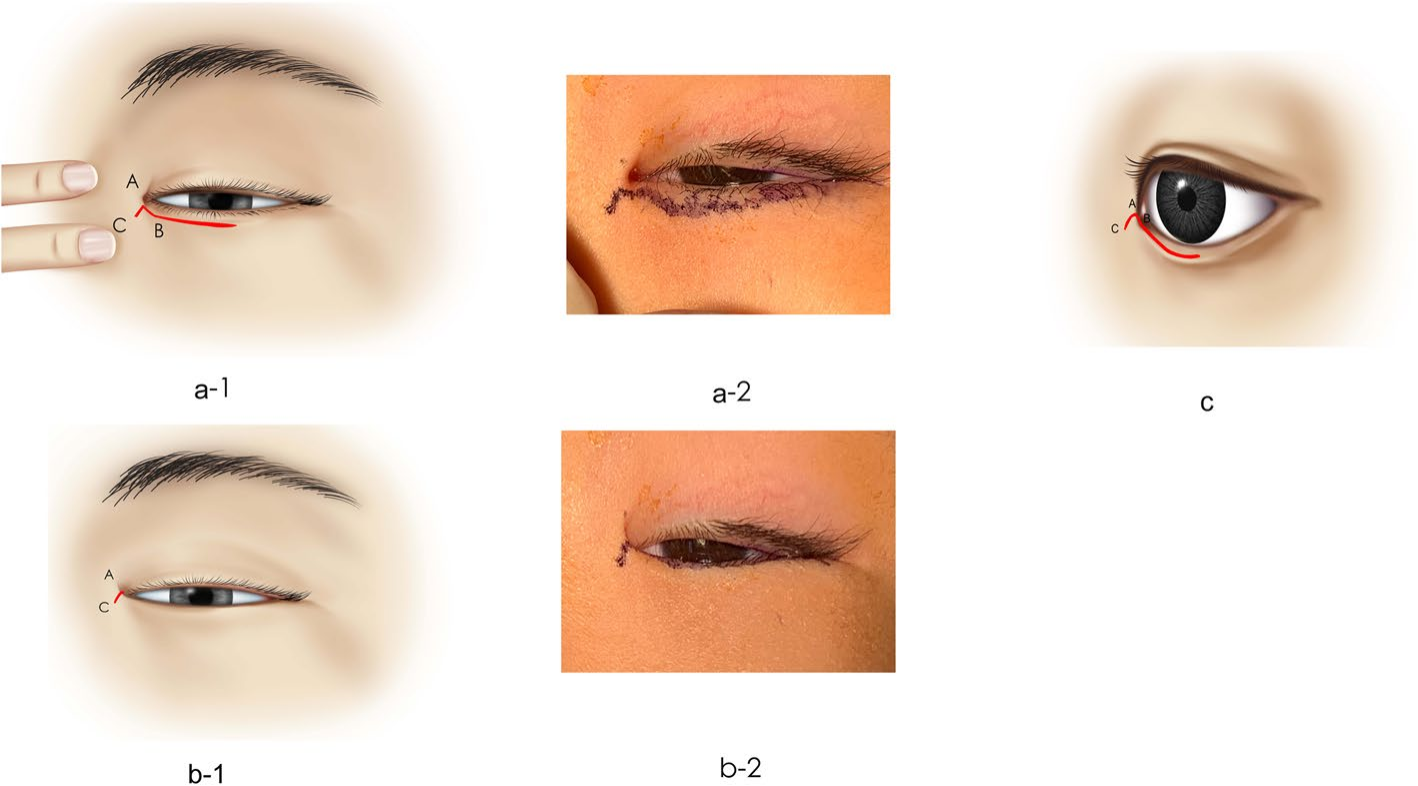
(a-1, b-1) Schematic diagrams and (a-2, b-2) photographs showing the preoperative incision design drawn under general anesthesia. The lines were marked after pulling the skin of the medial canthal area. The 20-mm–long incision began at the inner medial epicanthus and extended outside along the lower lid margin and was drawn 1.5–2 mm below the lash line. (b-1, c) Schematic diagrams showing the incision’s natural appearance under general anesthesia and the lateral appearance

**Fig. 4:**
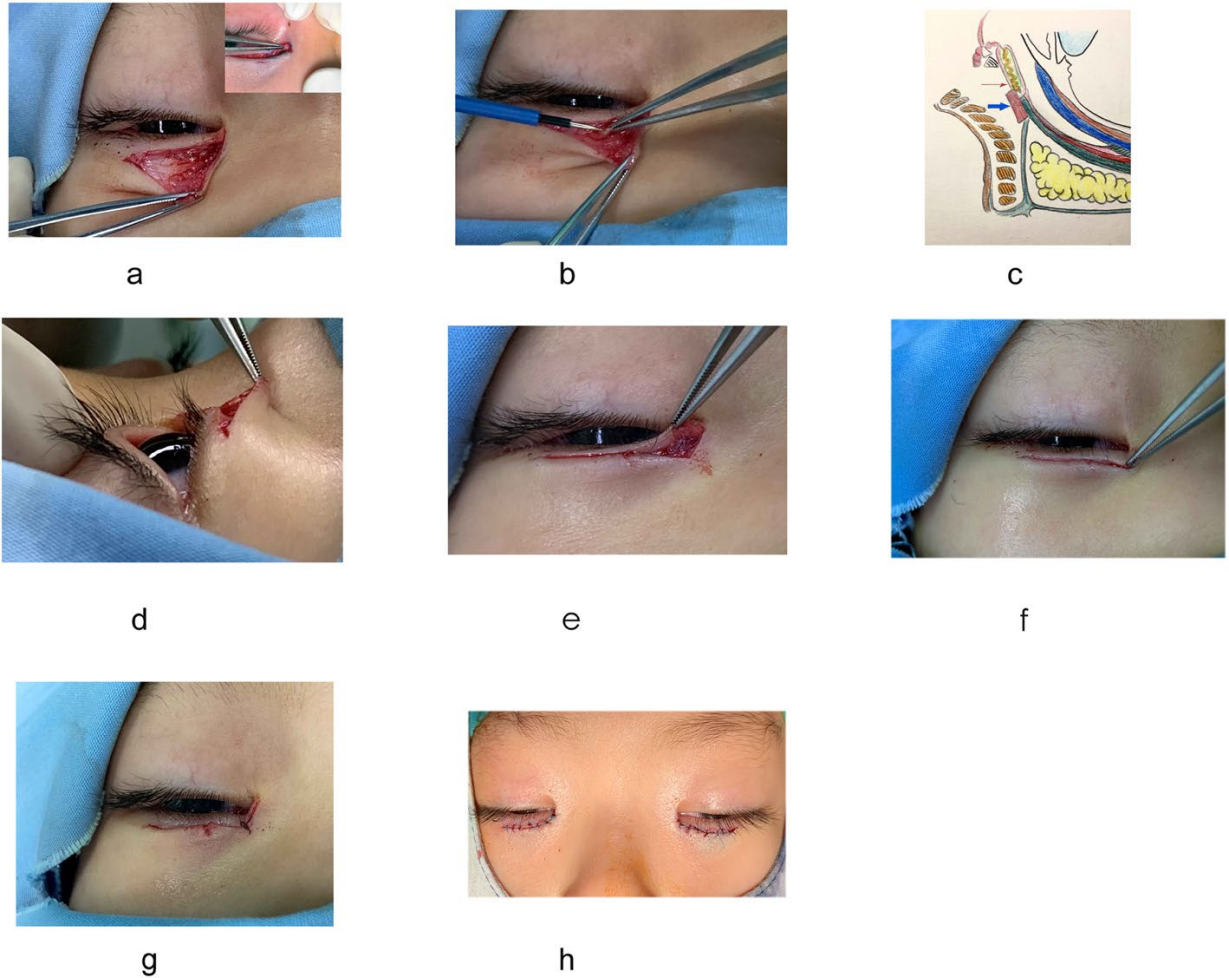
**a**, **b** Thermal cauterization was performed, and after removing and trimming the abnormal preseptal orbicularis oculi muscles and adhesions with the skin, tension was released when the dissection depth reached the medial canthal tendon. **c** Schematic diagram showing thermal cauterization. The large blue arrow indicates where cauterization was applied to create adhesions between the lower eyelid tractor and tarsus. The small red arrow shows where thermal contraction was applied to the tarsus (approximately 3–4 mm below the lid margin) if the lower eyelid lashes were not sufficiently reverted. **d** The lower eye lashes were inverted, so sutures were not needed to revert them. The epiblepharon was completely corrected from the lateral side view. **e**, **f** Image of the right eye shows decreased orbicularis tension; an inverted L-shape was obtained at the end of the procedure. The narrow medial epicanthus angle was open with upward upper skin traction. **g** The direction of the key medial suture. **h** Immediate postoperative appearance: The line was linear and smooth, and the appearance of the medial part of the lower eye lid was natural with no crease

After induction of general anesthesia, the lower eyelid surgery line was drawn 1.5 mm below the lash line, beginning at the inner medial epicanthus and extending to the temporal limbus (Fig. 3a-1, a-2). When the nasal skin is pulled medially to allow exposure, point A may be defined as the initial incision point, marked on the EFs along the epicanthus line at the level just above the caruncle. An oblique line was drawn medially from point A in an inferonasal direction to point C; the angle of line AC was ~ 45° (Fig. 3a-1, a-2), and the length to point C was ~ 1.5 mm. Schematic diagrams (Fig. 3b-1) and Fig. 3b-2 show the incision’s natural appearance under general anesthesia. Point B was marked on the lower eyelid incision line where the subciliary line met line AB, which was on the posterior surface of the medial canthal fold and was of the same length as AC. The final shape of the ABC markings shown in Fig. 3c resembling a triangular fish tail is based on a previous publication [10] with some modifications. Local anesthesia was administered using 1% xylocaine with 0.001% epinephrine to decrease bleeding. One piece of superficial orbicularis was carefully excised to expose the tarsal plate after cutting through the skin. The malpositioned orbicularis oculi muscle fibers underneath the EFs were then removed to decrease tension until the dissection depth reached the medial canthal tendon (Fig. 4a). Release of tension crossing the medial epicanthus usually normalizes the balance of the lower eyelid, to decrease the contractions of the skin which invert the eyelash to contact the eyeball.

After exposing the medial canthal tendon, we used a needle monopolar cautery instrument (Peng’s multifunctional operational dissectors; Shuyou Medical Equipment Co., Ltd, Zhejiang, China) with power set to 5–10 W at a contraction duration of 0.5 s/point until sufficient lash eversion was reached. Adhesions between the lower eyelid retractor and tarsus were created by thermal cauterization contraction [11]. The area of thermal contraction was proportional to the severity of lash inversion (Fig. 4b). If the eyelid lashes were not sufficiently rotated, additional thermal contraction was applied to the tarsus approximately 3–4 mm below the lid margin (Fig. 4c). To open the lower angle of the medial canthus, we sutured the residual edge (point B) to the point C (the most medial point) of the lower epicanthus (Fig. 4e and f). The first suture on the epicanthus was in the inferonasal direction (Fig. 4g) and the skin was closed using interrupted 7 − 0 nylon sutures (Fig. 4h), after the excess skin overlying the lower lid surgical line was precisely and carefully resected. Postoperatively, tobramycin antibiotic ointment was applied to the incision for two weeks until the sutures were removed.

Surgical success was defined as an absence of medial ciliary contact. Under-correction was defined as ciliary contact with only the medial conjunctiva one week after surgery. All the postoperative assessments were performed in an outpatient setting. Each patient was carefully monitored for surgical complications. The post-surgical result was observed using slit lamp examination. All patients received frontal and Lateral view photographs at pre-operative and each postoperative visit (1 month, 3 months, and 6 months after the surgery). The curvature of the lid margin and inner canthus and postoperative scarring were evaluated at the same time. Vancouver scar scale (VSS) scores were collected through clinical analysis by three independent doctors, to evaluate the incisional scars in the area of the lower eyelid and medial canthus.

Refraction and best-corrected visual acuity were measured pre- and at six months post-surgery. Refractive error was determined by cycloplegic retinoscopy, which was performed by retinoscopist in dim room light with a streak retinoscope, at least 30 min after instillation of 1% cyclopentolate hydrochloride using punctal occlusion with an interval of 10 min.

SPSS V25 for Windows (IBM Corporation, Armonk, NY) was used for statistical analysis. A paired t-test was applied to compare pre- and postoperative astigmatism in each patient, and two-sided *P* < 0.05 was considered statistically significant.

## Results

**Fig. 5:**
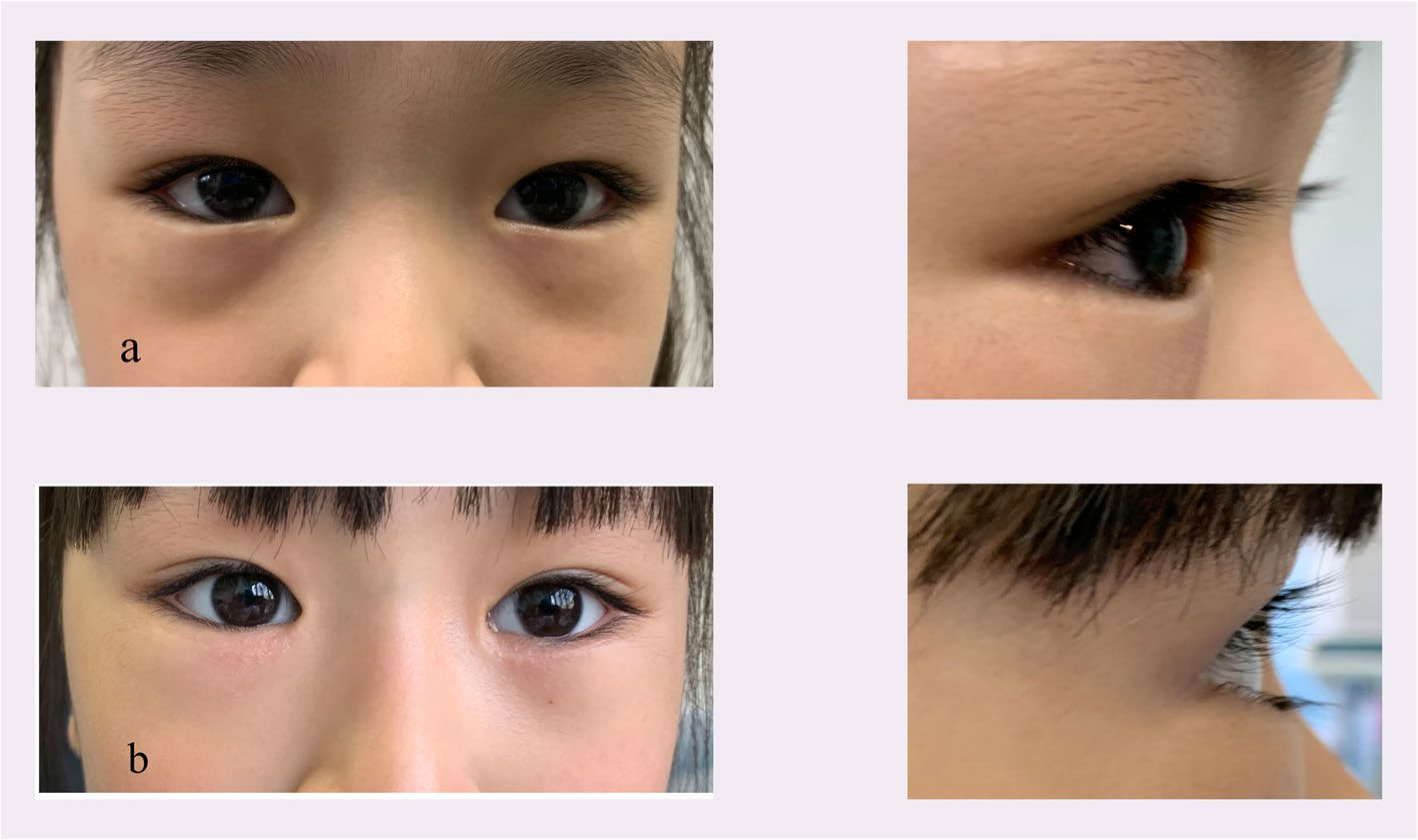
A 6-year-old girl with representative class III epiblepharon. **a** Preoperative image. **b** Six months postoperatively, the medial canthus angle was still open, and the eyelash direction was completely corrected

**Fig. 6:**
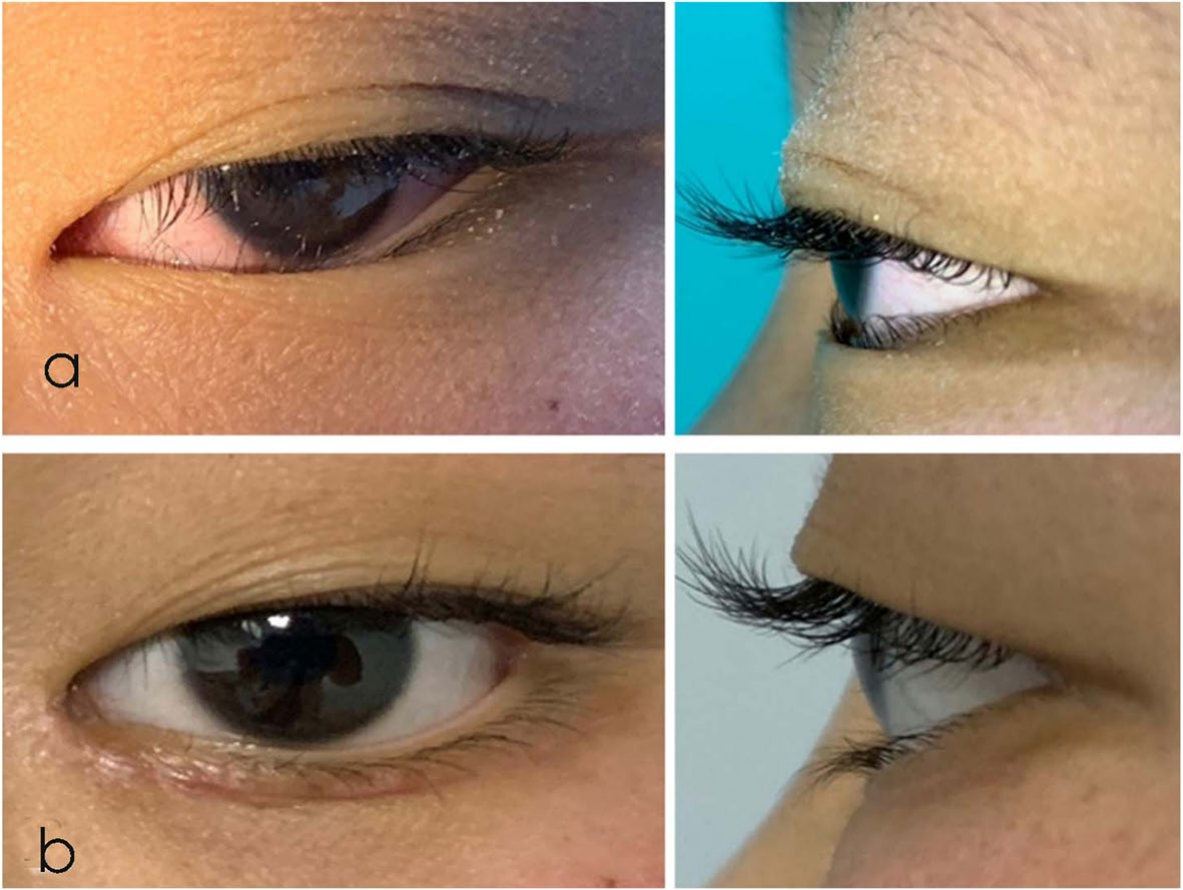
An 8-year-old boy with representative class IV epiblepharon. **a** Preoperative image. **b** Three months postoperatively, the cilia (especially in the medial part) were completely corrected; his pink eye and symptoms of irritation had resolved

Data from each eye of 53 Chinese children (29 female, 24 males; mean age: 5.3 ± 2.3 years) who had undergone minimal lower eyelid epicanthoplasty combined with thermal contraction were included. Fifty-one of the 53 patients (96.2%) had satisfactory correction of lower eyelid ciliary direction. The eyelashes were successfully everted, especially at the medial canthus area (Figs. 5 and 6 show representative cases), and no creases or obvious scars formed in the lower eyelids during the follow-up period. Recurrence was noted in one eye, and under-correction in two eyes (Table 1). Partial eyelash loss occurred in four children.

**Table 1:** Postoperative results

The classification of the epiblepharon (eye)	Well corrected, eye	Undercorrection, eye	Recurrence, eye
Class I (14)	14	0	0
Class II (28)	28	0	0
Class III (40)	40	0	0
Class IV (24)	21	2	1

At the preoperative visit, the mean (± standard deviation) spherical refractive errors of the right and left eyes were 0.14 ± 2.35 diopters (D) and 0.12 ± 2.24 D, respectively. Astigmatism > 1.25 D was found in 87/106 (82.1%) eyes. Most were with the rule (85 eyes, 80.19%); oblique astigmatism was found in only 10 (9.43%) eyes. The right and left eye preoperative astigmatism values were 1.77 ± 0.47 D and 1.87 ± 0.42 D, respectively. The mean astigmatism decreased significantly from 1.82 ± 0.45 D preoperatively to 1.43 ± 0.36 D at six months postoperatively (*P* < 0.05).

## Discussion

Asian populations have a high prevalence of bilateral epiblepharon, which can lead to ocular irritation and cornea erosion [1]. Although epiblepharon in some Asian children may resolve spontaneously with middle facial growth, surgery is indicated in cases with significant corneal irritation related to eyelash contact. Different surgical techniques have been described to correct this condition of which the modified Hotz procedure has been the most popular [12, 13]. However, due to anatomy of the medial epicanthus and the location at which the surgery is performed (lateral to the punctum), failure to entirely correct the medial cilia using this method contributes to the high recurrence rate of 9% [5]. Moreover, if insufficient skin is removed, the medial cilia may return to an inverted orientation [13]. Recent research shows that EFs are caused by malposition of the orbicularis oculi muscle passing over the medial canthal tendon. Therefore, some surgeons have recommended that epiblepharon in Asian subjects may be corrected by combining epicanthoplasty with a lower eyelid entropion procedure [14, 15]. Given the complexity of designing a procedure to modify an epicanthus flap, epicanthoplasty is not commonly used by Chinese ophthalmologists. Therefore, we developed a method which is easily designed and quickly performed. To reconstruct the lower eyelid with good stabilization and minimal scarring, we designed the flap to cross the lower EF, based on the lower triangle of a W-shaped flap [16], in contrast to other designs with a flap on the anterior surface of the EFs [10].

Previous article has emphasized that in the Hotz procedure or modified Hotz procedure the lower incisional line should be placed temporal to the inferior punctum [4]. However, using this approach it is difficult to completely correct the misdirected eyelashes at the most medial portion of the lower lid. In the present study, we performed surgery on the epicanthus medial to the inferior punctum, releasing the lower eyelid tension produced naturally by the anatomy of the medial epicanthus. This method facilitates the design and rearrangement of the medial lower eyelid in patients with epiblepharon (Fig. 3). After removing the hypertrophic orbicularis over the medial canthal tendon, the upper eyelid skin naturally lifted due to the anatomy of the epicanthus and the lower triangle incision became a straight line. The first suture on the epicanthus was in the inferonasal direction (Fig. 4g), where the residual medial edge was naturally sutured to the point C in our design. The inlay of the medial edge helped widen the medial angle and correct the most medial cilia. Moreover, it decreased the inward contraction force from the EF on the eyelashes and reshaped the epicanthus into an L-shape (Fig. 4e and f), which allowed it to connect with the lower eyelid skin naturally and linearly.

**Fig. 7:**
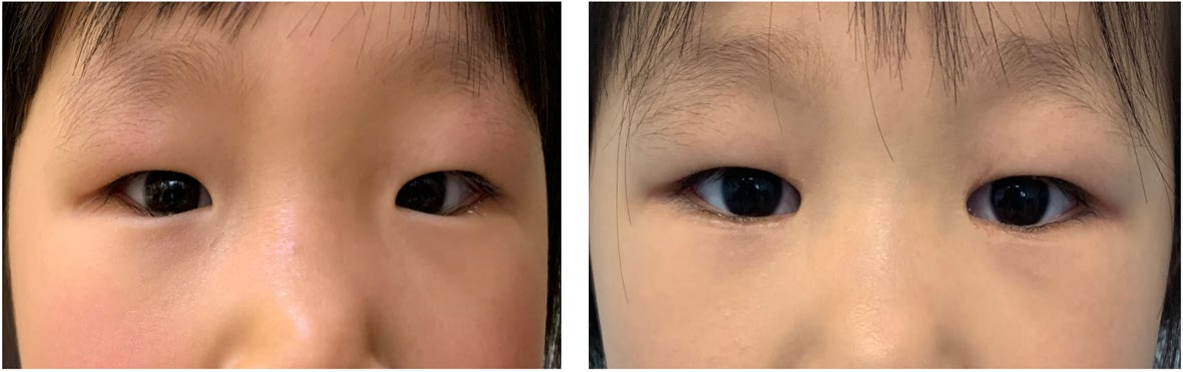
A 6-year-old girl underwent a modified re-draping method for epiblepharon correction. Her parents felt the surgery changed her appearance dramatically and made her look older

The goal of epicanthoplasty in adults is to improve cosmetic appearance by correcting the entire epicanthus. However, our existing methods seemed to be too aggressive for Asian children, and some parents complained that their children appeared old after surgery [17]. The novel method does not completely correct the whole epicanthus; it simply releases the tension of the lower epiblepharon and widens the epicanthus angle slightly. Postoperative appearance changes are less apparent than those in children who undergo epicanthoplasty using a modified re-draping method (Fig. 7).

One possible causative anatomical feature of epiblepharon is lack of adhesion between the lower eyelid retractors and the anterior lamella, which allows the skin and muscle to roll upwards. Previous studies have described surgical procedures creating a lower eyelid crease over the pre-existing fold, which have been widely used to treat epiblepharon [18–20]. However, for Asian subjects with no natural eyelid crease, lower eyelid creases are often cosmetically undesirable (Fig. 1). To avoid this problem, we cauterized the lower eyelid retractor and the tissue before tarsus to create a cicatricial barrier and minimize vertical strength (Fig. 4c) as described by Lee [11]. Thermal cauterization also enables controlled shrinkage of the lower eyelid retractor, sometimes including the orbital septum, to simply and effectively correct lower eyelid epiblepharon while preventing lid retraction complications [21].

Recent research shows that Chinese preschool children with lower eyelid epiblepharon have a higher risk of developing astigmatism than those without epiblepharon [22]. In our study, astigmatism > 1.25 D was found in 87/106 (82.2%) eyes. The mean astigmatism statistically significantly decreased from 1.82 ± 0.45 D preoperatively to 1.43 ± 0.36 D at 6 months after surgery; however, the change was not clinically significant. Some previous studies have also reported that epiblepharon correction surgery can significantly decrease astigmatism [23–25]. However, this finding is controversial since other studies have found no significant change in astigmatism after surgery in children with entropion and epiblepharon, with no influence of eyelid tension correction or healing of corneal erosions on astigmatism [26–28]. Therefore, anatomical studies and prospective case-control studies with larger samples are required to explore whether astigmatism is improved after surgery and how it changes over time.

In the present study, no cases of lid retraction or ectropion occurred during follow-up. Small scars were observed along the incisions in the first 1–3 months after the operation, but these were not present by 6 months postoperatively. However, four patients experienced cilia loss, so it is important to emphasize that thermal contraction should be performed with caution. The surgeon must pay special attention to protect the eyeball and eyelashes.

There are several advantages of our approach. (1) The simplicity of the design makes this procedure a very fast and easy way to achieve effective lash eversion. (2) Eyelid lashes are effectively rotated without significantly changing appearance or causing conspicuous scars. In this procedure, the skin incision was designed to open the EFs inferiorly, widening the lower angle of the epicanthus (especially in the medial conjunctival area) without affecting the thicker nasal skin. (3) By adjusting thermal contraction (e.g., power, duration, and area), the surgeon can control eyelash and lid margin rotation simply and rapidly, even without the rotational sutures if the epiblepharon is corrected as the Fig. 4d showed from the lateral side view.

The results of our study should be considered in the context of some limitations. First, the mean follow-up period was not sufficient to evaluate long-term results, especially considering that this was a pediatric population. Second, this was a non-comparative study, so it was not possible to compare the efficacy of this surgical technique with others.

## Conclusion

The minimal epicanthoplasty technique combined with thermal contraction is easy to design, time-efficient, safe, and effective to correct epiblepharon in Chinese children. It also delivers good cosmetic outcomes with relatively large eye contours without significant appearance changes. Finally, it avoids postoperative complications that may accompany the Hotz procedure.

## Data Availability

The datasets used and/or analyzed during the current study are available from the corresponding author on reasonable request.
